# New Non-Invasive Method for the Authentication of Apple Cultivars

**DOI:** 10.3390/foods11010089

**Published:** 2021-12-29

**Authors:** Elettra Barberis, Elia Amede, Francesco Dondero, Emilio Marengo, Marcello Manfredi

**Affiliations:** 1 Department of Translational Medicine, University of Piemonte Orientale, 28100 Novara, Italy; elia.amede@uniupo.it (E.A.); marcello.manfredi@uniupo.it (M.M.); 2 Center for Translational Research on Autoimmune and Allergic Diseases, University of Piemonte Orientale, 28100 Novara, Italy; emilio.marengo@uniupo.it; 3 Department of Sciences and Technological Innovation, University of Piemonte Orientale, 28100 Novara, Italy; francesco.dondero@uniupo.it

**Keywords:** food authentication, noninvasive analysis, gas chromatography, apples, pesticides

## Abstract

Food authentication is very important to protect consumers, sellers, and producers from fraud. Although several methods have been developed using a wide range of analytical techniques, most of them require sample destruction and do not allow in situ sampling or analysis, nor reliable quantification of hundreds of molecules at the same time. To overcome these limitations, we have developed and validated a new noninvasive analytical workflow for food authentication. The method uses a functionalized strip to adsorb small molecules from the surface of the food product, followed by gas chromatography–mass spectrometry analysis of the desorbed analytes. We validated the method and applied it to the classification of five different apple varieties. Molecular concentrations obtained from the analysis of 44 apples were used to identify markers for apple cultivars or, in combination with machine learning techniques, to perform cultivar classification. The overall reproducibility of the method was very good, showing a good coefficient of variation for both targeted and untargeted analysis. The approach was able to correctly classify all samples. In addition, the method was also used to detect pesticides and the following molecules were found in almost all samples: chlorpyrifos-methyl, deltamethrin, and malathion. The proposed approach not only showed very good analytical performance, but also proved to be suitable for noninvasive food authentication and pesticide residue analysis.

## 1. Introduction

Interest in and awareness of food traceability and authenticity is steadily increasing. At the same time, new analytical methods are urgently needed to help enforce regulations and detect illegal activities while protecting consumers from fraudulent and dangerous practices. The adulteration of food (i.e., adulteration or mislabeling of food) is not only one of the oldest illegal activities but also a widespread phenomenon in several countries. Therefore, new noninvasive methods are urgently needed to support consumers’ right to authenticity and detect food fraud.

To this end, the aim of the present research was to develop a new method for the noninvasive identification of fraud in agricultural products. In the past decade, several new methods for the chemical classification of food products have been developed, including spectroscopic techniques [1,2], gas chromatography and liquid chromatography coupled with mass spectrometry [3,4,5], direct analysis in real-time mass spectrometry (DART-MS) [6,7], and also electronic nose approaches [8]. All of these methods have always been coupled with multivariate techniques [9] and, more recently, with machine learning methods [10] to perform the automatic and increasingly accurate classification of foods based on chemical information.

Most of the available methods require sample destruction, and spectroscopic techniques, most of which have the great advantage of being noninvasive and portable, do not allow the accurate identification of the molecules present in a sample. To overcome these limitations, we have developed a noninvasive method to sample small molecules from food. To prove this concept, we used the method to characterize different apples to distinguish different varieties. Apple is one of the most commonly consumed fruits worldwide [11]. Over time, apple varieties have been selected based on their appearance, firmness, and storability. Apple cultivation is greatly influenced by geographical and climatic environments. In addition, apples are marketed throughout the year, so storage for long periods is widespread. Several laws and regulations have been enacted to ensure that labeling of products reflects their territorial origins [12]. In parallel, several scientific methods have been developed to certify the authenticity of apples and protect consumers and producers from fraud [13].

Most of the methods used for the characterization of apples involve sample destruction. For example, using a multi-element and multi-isotope approach, Aguzzoni et al. classified apples grown in different areas in northern Italy by linear discriminant analysis (LDA) with more than 96% accuracy [14]. Wang et al. used a microwave plasma torch (MTP) coupled with a linear ion trap time-of-flight mass spectrometer for food quality control such as checking for contamination, fraud, and authenticity [15]. Using infrared spectroscopy, Fernandez-Gonzalez et al. created an LDA model that can distinguish apples of different varieties [16]. In addition, a USB spectrometer with a wavelength range of 380 nm to 700 nm was used by Vincent et al. to build a classification model by pattern recognition to distinguish different apple varieties. The model successfully identified 93% of the samples [17].

In the present work, the development and validation of a new noninvasive method for the authentication of food products, especially apples, is presented. The method uses a functionalized strip to adsorb small molecules from the apple surface, followed by gas chromatography–mass spectrometry analysis of the desorbed analytes. The concentrations of the molecules are then used to identify markers of apple varieties or, in combination with machine learning techniques, to classify apples by geographical regions and variety. In addition, the method was tested to identify pesticide residues on apple peels.

## 2. Materials and Methods

### 2.1. Apple Samples

Apple samples were bought in different cities and from different resellers in order to account for the potential variability originating from different geographical provenience, different conservation methods, and different producers. Table 1 reports the details of the samples used: 44 apples were bought in 2019 in two cities (Alessandria and Novara, north Italy). The following varieties were selected: Ambrosia, Envy, Fuji, Gala, and Golden. The prices ranged from EUR 0.99 kg to EUR 2.99 kg.

### 2.2. Functionalized Strip Preparation

A special plastic-like film based on ethyl-vinyl acetate (EVA) (Ambra Polimeri srl, Agrate Brianza, MB, Italy) as a binder of ground AG 501 (Bio-Rad Laboratories, Hercules, CA, USA) mix-bed cation/anion exchange and C8 (Merck, Darmstadt, Germany) resins was prepared. Two types of resins were used in order to extend the molecule affinity of the strip. A mixture was made comprising 70% 1–10 μm size ground beads and 30% EVA. The melted EVA and Bio-Rad resins were extruded in the form of a thin film in the laboratory a week before use. The thickness of the film was 150–200 μm [18,19].

### 2.3. Non-Invasive Sampling

The plastic sheets were moistened in water before being applied to the surface of the apple. The strip was placed on the surface of the apple and gently pressed with the finger for 10 min. Then the support was transferred to a 1.5 mL Eppendorf microcentrifuge tube containing 300 µL ethanol for 15 min elution. Finally, the strip was removed, and the sample was dried with a stream of nitrogen.

### 2.4. Derivatization of the Small Molecules

The dried sample was processed through a two-stage derivatization. Briefly, 50 µL of methoxamine in pyridine (20 mg/mL) was added to the dried extract and then derivatized at 30 °C for 90 min. The sample underwent silylation with 50 µL of N,O-Bis(trimethylsilyl)trifluoroacetamide (BSTFA) at 37 °C for 30 min and then was centrifugated for 15 min at 20 °C at 14,500× *g* [20]. Finally, the tube content was transferred into a vial in which the internal standard hexadecane was added prior to the GC×GC-MS analysis. All the reagents and solvents were from Merck (Darmstadt, Germany).

### 2.5. Calibration Curves

The calibration curves of alpha-farnesene (Merck, Darmstadt, Germany) and Octanoic acid (Merck, Darmstadt, Germany) were carried out in a concentration range from 100 ppb to 1 ppm. The reproducibility and the recovery of the method were determined by depositing the standard solutions directly on the apple, then conserving the apple at 4 °C for one day, followed by extraction with the strip. Quality control samples (QC) containing spiked standards were also acquired before and after the analysis.

### 2.6. GCxGC-MS Analysis

For analysis of small molecules extracted from the apple surface, a LECO Pegasus BT 4D GCxGC/TOFMS set with a LECO dual-stage quad jet thermal modulator was used (LECO, Berlin, Germany). A previously described GCxGC-MS method was used [21].

### 2.7. Data Analysis

The chromatograms were acquired in TIC (total ion current) mode. If the ratio signal to noise, S/N, was smaller than 500, the analyte was discarded. For raw data processing, the ChromaTOF (LECO, Berlin, Germany) software was used. The library NIST MS Search 2.3 and the Fiehnlib were used for mass spectra identification. A monovariate (*p*-value < 0.05 and fold change > 1.3) and multivariate statistical analysis [22] was performed with the Metabolanalyst software 5.0 (www.metabo.metaboanalyst.org, accessed on 29 December 2021).

### 2.8. Machine Learning Analysis

We randomly divided the samples into two groups composed of 35 (training) and 9 (validation) samples. From the training cohort, we selected important metabolite features with recursive feature elimination using the random forest method. In the random forest analysis, 300 hundred trees were built using R package caret (version 4.6.14) with 20-fold cross validation repeated 5 times, and this whole framework was repeated 100 times. The selected important features were used for the random forest analysis of the independent test cohort (9 samples) [19].

## 3. Results

The ability to study molecules on the surface of apples, but more generally on all fruits and vegetables, can be of great value not only in developing new tools to fight food fraud but also in monitoring the freshness and quality of produce and the presence of illegal pesticides. Although several spectroscopic techniques have been developed to characterize foods, only mass spectrometry can effectively identify and quantify hundreds of molecules.

**Figure 1:**
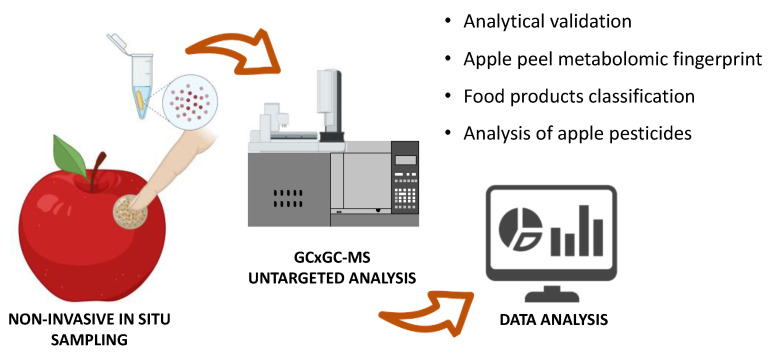
Experimental workflow of the methodology. Small molecules are sampled from the apple surface through a functionalized strip. The molecules are then eluted from the strip and analyzed using an untargeted metabolomic approach with a bidimensional gas chromatography system coupled with mass spectrometry. Markers of apple varieties are identified using both monovariate and multivariate statistical analysis. Finally, using classification algorithms, the apple varieties can be recognized. The analysis of pesticides from apple peel can also be performed.

One of the most critical problems in food analysis is sample destruction, which is required for the extraction of small molecules. Sample destruction precludes the ability to sell products and does not allow in situ sampling. To overcome these limitations, we developed a noninvasive method to extract small molecules from food (Figure 1). The method uses a functionalized strip based on functionalized films [18], which is directly contacted to the surface of the food. After the adsorption of the small molecules, the analytes were determined by gas chromatography–mass spectrometry. Finally, the abundances of the molecules and metabolomic fingerprints were used in combination with multivariate analysis and machine learning techniques to identify markers of geographical origin or varieties and to ensure the authenticity and traceability of food. Pesticide residue identification was also performed.

As a proof of concept, we developed and applied this method to the characterization of different apples to distinguish different varieties, leaving the product unchanged. The developed method allows the noninvasive extraction, identification, and quantification of the main components present on the surfaces of the apples. The method also allows the analysis of the molecules produced/released directly by the food over time.

### 3.1. Reproducible and Non-Invasive Sampling of Molecules

We first investigated the performance of the analytical method by analyzing the reproducibility of the sampling technique. Validation was performed using two standards (α-farnesene and octanoic acid). The standard solutions were applied to the surface of the apples and extracted using the developed method after complete evaporation of the solvent. The two molecules were then analyzed by gas chromatography–mass spectrometry to obtain a calibration curve of the standard solutions. The concentrations of the two molecules were in the following range: from 100 ppb to 1 ppm (100 ppb, 250 ppb, 500 ppb, 750 ppb, 1 ppm, 10 ppm). The regressions coefficients obtained were 0.99 and 0.98 for α-farnesene and octanoic acid, respectively. The limit of detection (LOD) and limit of quantification (LOQ) were 150 ppb and 200 ppb for α-farnesene, and 200 ppb and 250 ppb for octanoic acid, respectively. The recovery of the method was also tested at two values (250 ppb and 1 ppm): the results showed a low recovery rate of 9.6% and 10.4% for α-farnesene and 12.3% and 14.1% for octanoic acid, respectively. Finally, the inter-day and intra-day reproducibilities of the method were 5.6% and 4.1% for α-farnesene and 7.5% and 6.3% for octanoic acid, respectively. The recovery of the method is very low because the technique was developed as noninvasive. However, the recovery could be potentially improved by changing the mixture of the resin of the functionalized film. The functionalized films were made using EVA polymer, which is particularly suitable for this application because it can be dissolved in cyclohexane and thus it can easily be mixed with the resins. As adsorption resins, AG 501 and C8 were selected. The first is a strong cation and anion exchange resin, while C8 is similar to C18 but it is slightly more polar. These resins were chosen in order to capture a wider range of different molecules.

The overall data showed very good performance in terms of the validation of the method. Although the recovery of the molecules was low, the method was reproducible, accurate and precise.

### 3.2. Small Molecules and Apple Surface Variability

To explore the potential use of the method for the noninvasive analysis of apples and to investigate the analytical properties of the technique, we used the strip to adsorb small molecules from three different areas of the same apple. The small molecules extracted with the strip were then subjected to untargeted metabolomic analysis using two-dimensional gas chromatography coupled to a mass spectrometer. The analysis allowed the identification of nearly 800 molecules (Supplementary Table S1), including esters, alcohols, sugars, terpenes, fatty acids, alkanes, and aldehydes. Interestingly, among these molecules were several apple markers such as α-farnesene, mannopyranose, glucopyranose, mannose, glucose, galactopyranose, talose, allopyranose, talopyranose, benzyl alcohol, isobutanol, 2-heptanol, octanoic acid, 1,3-propanediol, 2-nonenal, hexanoic acid hexyl ester, phenoxyethanol, cyclohexanol, and 1-octanol 2-butyl.

The surface variability of the apple was determined by calculating the coefficient of variation (CV%) between three replicates performed on different sites of the same apple. The results showed that the intrinsic variability of the apple surface was very low: 40% of the total quantified molecules had a CV% of less than 30% (average CV% = 16%) and the overall average CV% was 42%. This result indicates that the surface of the apple was sufficiently homogeneous from a chemical point of view, suggesting the potential use of the surface sampling method for the analysis of apples.

### 3.3. Variety-Based Metabolomic Fingerprinting of Apples

To investigate the influence of different apple cultivars on the surface/peel metabolome, the developed method was applied to the analysis of 44 different apples. The most common varieties, including Fuji, Gala, Golden, Envy, and Ambrosia, were selected. To obtain a representative sampling, the apples were purchased in two different cities and in 12 different fruit retailers, GDO and small markets. In addition, four red apple varieties (Fuji, Gala, Envy, and Ambrosia) and only one yellow variety (Golden) were included in the study to understand whether the method could also distinguish similar varieties (the red). The full details of the samples are given in Table 1.

The metabolomic analysis of the extracted metabolites allowed the identification of hundreds of molecules, as reported in Section 3.2. The metabolomic profiles of the apples were analyzed using univariate and multivariate analysis to determine quantitative differences between cultivars.

**Figure 2:**
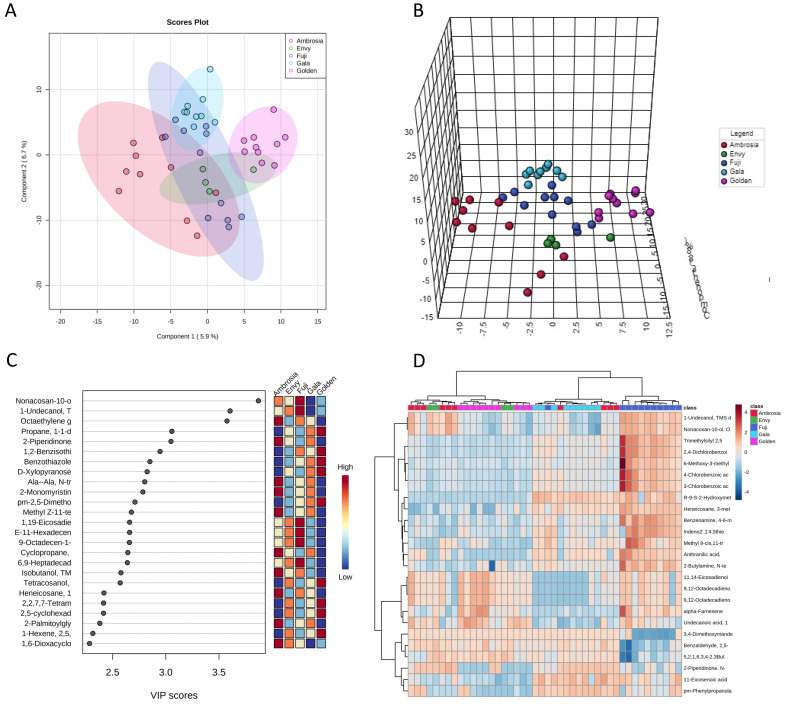
Metabolomic fingerprinting of apples. Partial least squares discriminant analysis (PLS-DA) of the apple metabolome of Ambrosia (red dots), Envy (green dots), Fuji (blue dots), Gala (light blue dots), and Golden (purple dots). The five groups are well separated (**A**,**B**). Important features identified by PLS-DA (**C**): colored boxes indicate the most predictive or discriminative features in each group (red, high; blue, low). Hierarchical clustering heatmap of surface molecules (**D**).

First, we examined the profiles for the presence of unique signatures associated with the apple cultivars. As shown in the score plots in Figure 2A,B, partial least square discriminant analysis (PLS-DA) clearly showed the presence of metabolomic profiles associated with apple cultivars. Some samples were more homogeneous and grouped together: this was the case with Gala and Golden—the former being a cross between the latter and the cultivar Kidd’s Orange Red—whereas in others, the variability was higher (Ambrosia and Fuji). The most predictive or discriminatory traits, potentially useful in classifying the samples, were also determined by the variable of importance in projection (VIP) score. The VIP score summarizes the most important molecules responsible for the reported molecular variations in the analyzed apples (Figure 2C). The hierarchical clustering heatmap of surface molecules in Figure 2D not only shows the presence of a common metabolome in apples of the same cultivars but the ability of the method to detect it.

### 3.4. Biomarkers of Apple Cultivars

**Figure 3:**
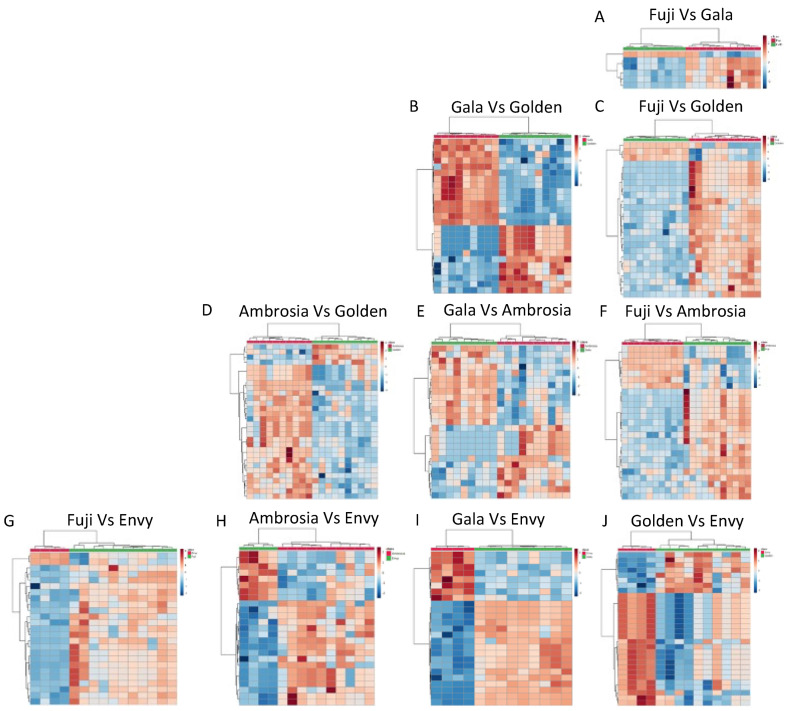
Hierarchical clustering heatmaps made using the top modulated molecules in the following comparisons: Fuji versus Gala (**A**), Gala versus Golden (**B**), Fuji versus Golden (**C**), Ambrosia versus Golden (**D**), Gala versus Ambrosia (**E**), Fuji versus Ambrosia (**F**), Golden versus Envy (**G**), Ambrosia versus Envy (**H**), Gala versus Envy (**I**), and Fuji versus Envy (**J**).

The identification of biomarkers of apple varieties is very important to detect food fraud. As reported in the previous subsection, a multivariate analysis showed the presence of a metabolic fingerprint that correlated with the different cultivars. Here, we examine the presence of specific molecules that might be able to distinguish the samples. We directly compared the abundance of the molecules among all five groups. Figure 3 shows the hierarchical clustering heatmap of all the comparisons between the different varieties performed using the most modulated molecules for each analysis. The results show that there are several molecules that vary in abundance between the apple groups. Notably, very little variability was observed between apples of the same variety. Apples were purchased in different cities and from different distributors to account for the potential variability caused by different geographic origins, different preservation methods, and different growers. Our data showed that all of these factors minimally affected the surface metabolome, indicating the potential utility of this method for apple traceability and authentication. This is particularly evident when looking at specific biomarkers. The full list of molecules with different abundances between the different apple groups can be found in Supplementary Table S2. In Supplementary Figure S1 representative two-dimensional chromatograms are also reported.

**Figure 4:**
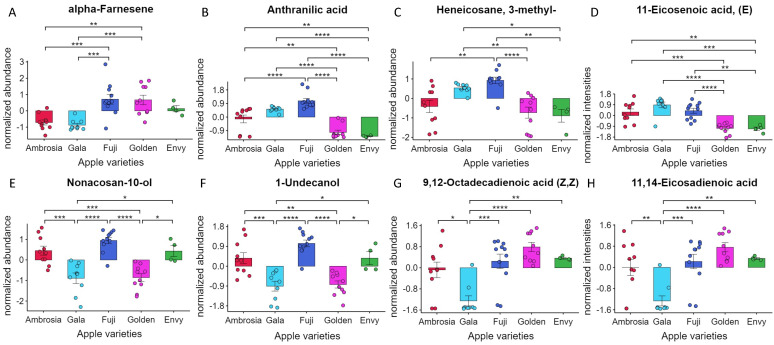
Boxplots of apple varieties markers. Relative abundances of alpha-Farnesene (**A**), Anthranilic acid (**B**), Heneicosane, 3-methyl- (**C**), 11-Eicosenoic acid (**D**), Nonacosan-10-ol (**E**), 1-Undecanol (**F**), 9,12-Octadecanoic acid (Z,Z) (**G**) and 11,14-Eicosadienoic acid (**H**) of the apple metabolome of Ambrosia (red dots), Envy (green dots), Fuji (blue dots), Gala (light blue dots), and Golden (purple dots). *p*-value < 0.05 = *, *p*-value < 0.01 = **, *p*-value < 0.001 = ***, *p*-value < 0.0001 = ****.

Several hundred compounds have been identified as aroma compounds from different apple cultivars [23], and some of them have also been associated with the sensory quality of apple fruit [24]. As shown in Figure 4, several molecules can be used to distinguish among different apple varieties. For example, the content of alpha-farnesene (Figure 4A), a terpenoid present in almost all plant species, was higher in Fuji, Golden, and Envy, allowing the distinction between Ambrosia and Gala. Terpenoids play a crucial role in plant metabolism [25]. Plants produce many terpenes, mainly branched acyclic sesquiterpenes, namely alpha-farnesene. Terpenes are a key factors in plant trophic interactions. Alpha-farnesene is associated with ripening and indeed is correlated with the amount of ethylene: its content increases during the ripening process. It has also been shown to play an important role in the defense against pathogens.

Another interesting marker was anthranilic acid (Figure 4B), the precursor of the ester methyl anthranilate, which is characteristic of the fruity grape scent. The ANOVA showed that the amount of anthranilic acid was correlated with apple cultivars: Golden and Envy had lower levels than Ambrosia, Gala, and Fuji. Krokida et al. used gas chromatography to study the aroma of apples during freezing and during convective drying at different temperatures and moisture levels. They found approximately 40 molecules that are representative components of apple aroma, including methyl anthralinate [26].

Waxes are one of the main components of the surface layers in apples and in all plants exposed to the atmosphere. This lipid material serves as a natural barrier to the environment [27]. Our data showed that heneicosane-3-methyl (Figure 4C) can be used to distinguish Fuji from Ambrosia, Golden, and Envy, and to distinguish Gala from Golden and Envy. Furthermore, 9,12-octadecadienoic acid was less abundant in Gala than in the other groups (Figure 4G).

Another interesting molecule is 11,14-eicosadienoic acid, which was less abundant in Gala apples (Figure 4H). The abundance of palmitic acid (C16:0) and oleic acid (C18:1) depends on the first stage of cuticular wax biosynthesis [28]. As mentioned by Klein et al. [29], 11,14-eicosadienoic acid (C20:2) comes from the elongation of C16:0 and C18:0 due to the synthesis of very long-chain fatty acids, producing fatty acids from C20 to C34.

Finally, alcohols are strongly associated with the smell of fruits. 1-Undecanol, a compound belonging to the fatty alcohol class, is formed by the reduction of the analogous aldehyde and is partially responsible for the typical fruit odor. Its content was higher in Ambrosia, Fuji, and Envy (Figure 4F). Moreover, 10-nonacosanol (Figure 3E), already proposed as a marker for the Golden Delicious cultivar [30], showed the same behavior as 1-undecanol.

### 3.5. Classification of Apple Varieties by Chemical Fingerprinting

We further investigated whether the chemical fingerprints of small molecules obtained from different apple varieties could be used for automatic classification using a machine learning approach. Envy apples were excluded because only four samples were available. Random forest was used to build a model based on metabolomic data randomly selected from 80% of the samples for each apple group. The model was then externally validated on the remaining 20% of the samples and achieved an average area under the curve of 1 (100 iterations) with 100% correct classification. Interestingly, among the most important features selected by the algorithm for classification, the best biomarkers identified by monovariate analysis were alpha-farnesene, anthrallinic acid, 1-undecanol, and heneicosane 3-methyl.

These results suggest that our sampling technique using the strips, combined with a gas chromatography–mass spectrometry method and a machine learning approach, can be used for noninvasive classification of apple cultivars, thus counteracting food fraud in the agricultural products field. We have also obtained some preliminary results on other food products such as tomatoes, oranges, and pears: the method was suitable for the adsorption of the surface molecules from these food products.

### 3.6. Non-Invasive Identification of Pesticides

Although the use of pesticides in agriculture has improved farm productivity, residues may persist in agricultural products and contribute to overall dietary pesticide exposure. Pesticide residues are often removed from produce because of growing concern about the potential hazards they pose to food safety and human health. Gas chromatography and liquid chromatography are widely used methods for detecting pesticides in agricultural products. However, these methods require complicated pretreatment and are sample destructive [31]. Recently, several spectroscopic methods have been developed for the rapid and nondestructive detection of chemical contaminants in complex food matrices [32]; and among these tools, surface-enhanced Raman spectroscopy (SERS), which is an integration of nanotechnology and the Raman spectroscopic technique, proved to be one of the most promising [33]. However, these methods have some limitations, including the need for expensive instruments that are not widely used in quality control laboratories, and the impossibility of performing untargeted analysis or multiresidue analysis with hundreds of molecules.

**Figure 5:**
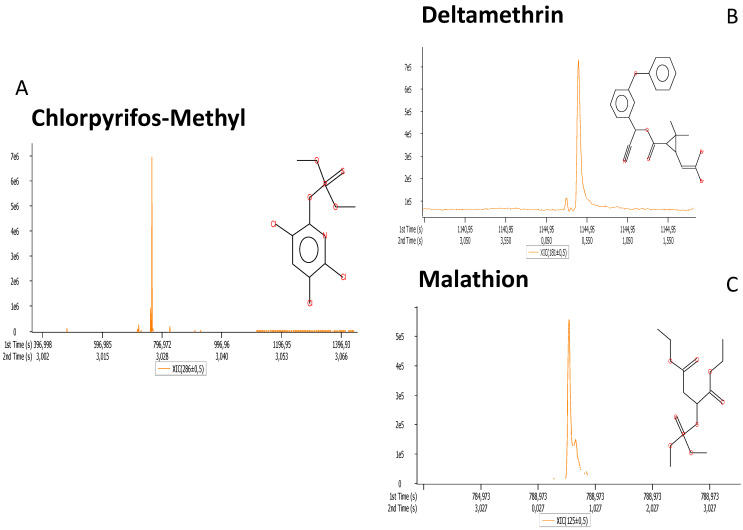
Pesticide residues identified on apple surfaces using the developed method: chlorpyrifos-methyl (**A**), deltamethrin (**B**), and malathion (**C**).

We then decided to investigate whether our developed approach was also suitable for noninvasive analysis of pesticides from apple peels. An analysis of the 44 apple samples revealed the presence of three pesticides: chlorpyrifos-methyl, deltamethrin, and malathion (Figure 5). These molecules were identified in almost all samples: chlorpyrifos-methyl was present in about 75% of the samples, deltamethrin was identified in all samples, while malathion was detected in about 88% of the analyzed samples. In agreement with our data, the 2018 “Official Report on Product Residues in Food” of the Italian Ministry of Health shows that chlorpyrifos-methyl and deltamethrin are the substances most frequently detected in Italian agriculture in absolute terms [34]. In 2020, the European Union banned the use of chlorpyrifos and chlorpyrifos-methyl with the recent Regulations 2020/17 and 2020/18, published in the *Official Journal of the European Union* on 13 January 2020 [35]. The use of malathion as a plant protection product was revoked and allowed in the EU until January 2020 because it was classified as a probable or possible human carcinogen by the WHO [36].

Although no quantitative analysis was performed, these results suggest that the developed method is suitable for the analysis of pesticides from agricultural products. This is also confirmed by the analytical performances obtained with other molecules in Section 3.1 and Section 3.2. One of the main advantages of the present approach is the possibility of performing a noninvasive and in situ sampling, leaving the product unchanged. This possibility can open up the development of new approaches to food quality control.

## 4. Conclusions

In the present study, we developed and applied a new noninvasive method for the authentication of agricultural food products. The method uses a functionalized strip to adsorb small molecules from the surface of the sample, leaving the product unchanged. The molecules were then analyzed by gas chromatography–mass spectrometry to obtain a metabolic fingerprint. We have fully validated and applied the method for the discrimination of five different apple varieties. Molecular concentrations obtained from the analysis of 44 apples were used to identify markers of apple cultivars and perform cultivar-based classification. The overall reproducibility of the method was very good, showing a good coefficient of variation for both the targeted and untargeted analyses. The approach was able to correctly classify all samples correctly. In addition, we also investigated the presence of pesticides on apple peels. We identified three molecules in almost all samples, namely chlorpyrifos-methyl, deltamethrin, and malathion.

The developed method offers two main advantages. First, the samples do not need to be destroyed; this is very important because it does not exclude the possibility of selling the product. Second, sampling can be done directly on-site, which is highly suitable for conducting large-scale studies. However, the limitations include the time for sampling, which can be between 10 and 15 min, and the possibility of extracting a wide range of molecules, which can be limited by the solvent used (water) and the affinity of the strip for the molecules. In conclusion, the proposed approach shows excellent analytical performance and is also suitable for noninvasive food authentication and pesticide residue analysis.

## Figures and Tables

**Table 1:** Features of apple samples used in the present study.

Cultivar	Number of Samples	Price Range (EUR/kg)
Ambrosia	10	2.20–3.40
Envy	4	3.60–4.20
Fuji	11	0.99–2.99
Gala	9	1.09–3.40
Golden	10	1.49–2.19

## Data Availability

Not applicable.

## Supplementary Materials

The following are available online at https://www.mdpi.com/article/10.3390/foods11010089/s1, Table S1: Identified molecules on apple surface, Table S2: Molecules with different abundances between apple groups, Figure S1: Representative two-dimensional chromatograms for Fuji, Gala, Ambrosia, Golden, and Envy apples.
