# Supplementary material for: New Non-Invasive Method for the Authentication of Apple Cultivars
Source: Foods. 2021 Dec 29;11(1):89. doi: 10.3390/foods11010089 (PMC8750361; doi:10.3390/foods11010089)
Supplement: Supplementary file 1 [file foods-11-00089-s001.zip › foods-1525424-supplementary.pdf]

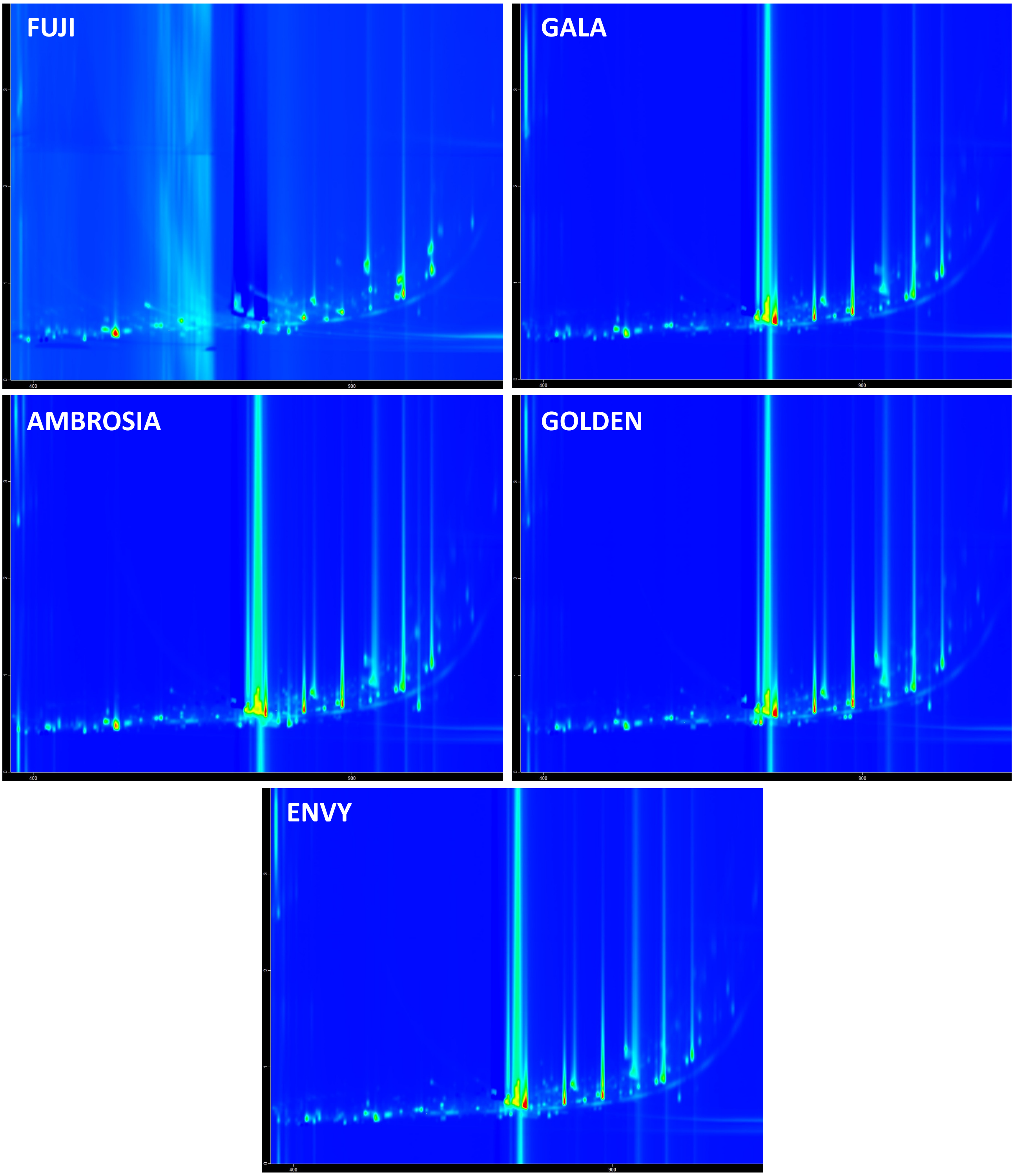


Figure S1. Representative two-dimensional chromatograms for Fuji, Gala, Ambrosia, Golden and Envy apples.

Table S1. Identified molecules on apple surface.

| Molecules |
|---|
| (2,5-Dimethoxyphenyl)acetone |
| (2-Aziridinylethyl)amine |
| (2E,6E)-3,7,11-Trimethyldodeca-2,6,10-trien-1-yl stearate |
| (3a?,4?,7?,7a?)-3a,4,7,7a-Tetrahydro-4,7-methano-1H-indene |
| (4,4-Dimethyl-5-methylene-4,5-dihydro-3H-pyrrol-2-yl)-(4,4-dimethyl-5-methylene-pyrrolidin-2-ylidene)-acetonitrile |
| 2,5-Dimethoxy-4-Iodoamphetamine, N-trimethylsilyl- |
| 3,4,5-Trimethoxyamphetamine, N-(trimethylsilyl)- |
| 4-Methylephedrine, N-trimethylsilyl-, trimethylsilyl ether |
| 4-Methyl-N-ethyl-norephedrine, N-trimethylsilyl-, trimethylsilyl ether |
| MBDB, N-trimethylsilyl- |
| N-Ethylcathinone ephedrine, N-trimethylsilyl-, trimethylsilyl ether |
| Phenylpropanolamine, N-trimethylsilyl-, trimethylsilyl ether |
| p-Methoxyamphetamine, N-trimethylsilyl- |
| (R)-(-)-Phenylephrine, N-trimethylsilyl-, bis(trimethylsilyl) ether |
| (R)-9-[(S)-2-(Hydroxymethyl)pyrrolidin-1-yl]-3-methyl-3,4-dihydro-2H-benzo[b][1,4,5]oxathiazepine 1,1-dioxide |
| (S,E)-8,12,15,15-Tetramethyl-4-methylenebicyclo[9.3.1]pentadeca-7,11-diene |
| (Z)-2-(Heptadec-10-en-1-yl)-4,5-dihydro-1H-imidazole |
| (Z)-Docos-13-enamide, N-TMS |
| (Z)-Docos-9-enenitrile |
| [1,1'-Biphenyl]-4,4'-diamine, 3,3',5,5'-tetramethyl- |
| 1-(1-Butoxy-2-propoxy)-2-propanol, TMS derivative |
| 1-(2,4-Dimethoxyphenyl)-propan-2-one |
| 1-(2,5-Dimethoxyphenyl)-1-[(trimethylsilyl)oxy]propan-2-amine |
| 1-(2-Methoxy-1-methylethoxy)-2-propanol, TMS derivative |
| 1-(Ethoxycarbonylmethyl)pyridinium bromide |
| 1,1,3,3,5,5,7,7-Octamethyl-7-(2-methylpropoxy)tetrasiloxan-1-ol |
| 1,11-Dodecadiene |
| 1,15-Hexadecadiene |
| 1,19-Eicosadiene |
| 1,2,3-Butanetriol, 3TMS derivative |
| 1,2-Benzenedicarboxylic acid, bis(2-ethylhexyl) ester |
| 1,2-Benzenedicarboxylic acid, bis(2-methylpropyl) ester |
| 1,2-Benzenedicarboxylic acid, dinonyl ester |
| 1,2-Benzenediol, O-(4-methoxybenzoyl )-O'-(2-furoyl)- |
| 1,2-Benzisothiazole |
| 1,2-Cyclohexanedicarboxylic acid, 2-ethylhexyl nonyl ester |
| 1,2-Cyclohexanedicarboxylic acid, 2-methylpent-3-yl nonyl ester |
| 1,2-Cyclohexanedicarboxylic acid, cyclohexylmethyl nonyl ester |
| 1,2-Cyclohexanedicarboxylic acid, dinonyl ester |
| 1,2-Cyclohexanedicarboxylic acid, heptadecyl 2-methylcyclohexyl ester |
| 1,2-Ethenediol, 2TMS derivative |
| 1,3,5-Trioxepane |
| 1,3-Bis(trimethylsilyloxy)pentane |
| 1,3-di-iso-propylnaphthalene |
| 1,3-Dioxolane, 2-butyl- |
| 1,3-Dioxolane, 2-heptyl- |
| 1,3-Dipentyl-heptabarbital |
| 1,3-Diphenyl-2-azafluorene |
| 1,3-Propanediol, 2TMS derivative |
| 1,4-Benzenedicarboxylic acid, bis(2-ethylhexyl) ester |
| 1,4-Butanediol, 2TMS derivative |
| 1,5-Anhydroglucitol, 4TMS derivative |
| 1,5-Heptadiene, 3,4-dimethyl- |
| 1,6-Dioxacyclododecane-7,12-dione |
| 1,8,11-Heptadecatriene, (Z,Z)- |
| 10,18-Bisnorabieta-8,11,13-triene |

| |
|---|
| 10H-Phenoxaphosphine, 2-chloro-8-ethyl-10-hydroxy-, 10-oxide |
| 10-Undecen-1-ol, TMS derivative |
| 10-Undecen-1-yl acetate |
| 11,14-Eicosadienoic acid |
| 11,14-Eicosadienoic acid, methyl ester |
| 11-Eicosenoic acid, (E)-,TMS derivative |
| 11-Hexacosyne |
| 11-Hydroxy-.DELTA.-9-tetrahydrocannabinol, bis(trimethylsilyl) ether |
| 11-Octadecynenitrile |
| 13-Bromotetradecanoic acid |
| 13-Docosen-1-ol, (Z)- |
| 13-Docosenamide, (Z)- |
| 16-Octadecenoic acid, methyl ester |
| 18-Methyl-eicosane-1,2-diol, isopropylidene derivative |
| 19-Norepietiocholanolone acetate |
| 1-Aminocyclopentanecarboxylic acid, 3-chloropropoxycarbonyl-, tridecyl ester |
| 1-Butoxy-2-propanol, TMS derivative |
| 1-Chloroundecane |
| 1-Cyclohexylethanol, TMS derivative |
| 1-Decanol, 2-methyl- |
| 1-Decene |
| 1-Deoxypentitol, 4TMS derivative |
| 1-Diphenyl(tert-butyl)silyloxy-4-methoxybenzene |
| 1-Dodecanol |
| 1-Dodecanol, 2-hexyl- |
| 1-Dodecanol, TMS derivative |
| 1-Ethyl-2-dimethyl(octyl)silyloxycyclohexane |
| 1-Heneicosanol |
| 1-Hexacosene |
| 1-Hexadecanol, 3,7,11,15-tetramethyl- |
| 1-Hexadecanol, TBDMS derivative |
| 1-Hexadecanol, TMS derivative |
| 1-Hexanone, 5-methyl-1-phenyl- |
| 1-Hexene, 2,5,5-trimethyl- |
| 1H-Imidazole, 2-heptadecyl-4,5-dihydro- |
| 1H-Imidazole, 4,5-dihydro-2-hexadec-9Z-enyl- |
| 1H-Indene, 1-ethylidene- |
| 1H-Indene, 1-hexadecyl-2,3-dihydro- |
| 1H-Indene, 1-methylene- |
| 1H-Quinolin-2-one, 5,8-dimethoxy-1,4-dimethyl- |
| 1-Methyl-5-mercaptotetrazole |
| 1-Mono-isobutyrin, 2TMS derivative |
| 1-Monolinolein, 2TMS derivative |
| 1-Monomyristin, 2TMS derivative |
| 1-Monopalmitin, 2TMS derivative |
| 1-Naphthalenecarboxamide, N-(3-chlorophenyl)- |
| 1-Naphthamide, N-(4-chlorophenyl)- |
| 1-Octacosanol, 2,4,6,8-tetramethyl-, (all-R)- |
| 1-Octadecanol, TBDMS derivative |
| 1-Octadecanol, TMS derivative |
| 1-Octanol, 2,2-dimethyl- |
| 1-Octanol, 2-butyl- |
| 1-Octanol, 3,7-dimethyl- |
| 1-Octene, 6-methyl- |
| 1-Pentanol, 2-ethyl-4-methyl- |
| 1-Pentene, 2,4,4-trimethyl- |
| 1-Phenyl-1,2-ethanediol, 2TMS derivative |
| 1-Propanol, TMS derivative |

1-Propene-1-thiol
1-Tetracosene
1-Tetradecene
1-Tricosene
1-Undecanol, TMS derivative
2-(2,5-Dimethoxy-phenyl)-propionaldehyde
2(3H)-Furanone, 5-hexyldihydro-
2-(4'-Methoxyphenyl)-2-(3'-methyl-4'methoxyphenyl)propane
2,2,7,7-Tetramethyltricyclo[6.2.1.0(1,6)]undec-4-en-3-one
2,2-Dimethyl-3-heptene trans
2,2'-Dithiobisethanol, 2TMS derivative
2,2'-Heptamethylene-di-2-imidazoline
2,3,4-Trihydroxybutyric acid tetrakis(trimethylsilyl) deriv., (, (R*,R*)-)
2,3-Butanediol, 2TMS derivative
2,3-Dihydro-1H-2-isopropylcyclopenta[b]quinoxaline
2,3-Dihydroxypropyl icosanoate, 2TMS derivative
2,3-Dimethoxyphenol, TMS
2,4,6,8-Tetramethyl-1-undecene
2,4,6-Tris(1,1-dimethylethyl)-4-methylcyclohexa-2,5-dien-1-one
2,4,7,9-Tetramethyl-5-decyne-4,7-diol, 2TMS derivative
2,4-Dichlorobenzoic acid, TMS derivative
2,4-Dihydroxybenzoic acid, 3TMS derivative
2,4-Di-tert-butylphenol
2,4-Thiazolidinedione
2,5-cyclohexadiene-1,4-dione, 2-(1,1-dimethylethyl)-5-(2-methyl-2-propen-1-yl)-
2,5-Cyclohexadiene-1,4-dione, 2,6-bis(1,1-dimethylethyl)-
2,5-Dihydroxy-4-methoxyacetophenone
2,5-Dimethoxy-4-methylamphetamine, N-(trimethylsilyl)-
2,5-Dimethoxybenzoic acid, TMS derivative
2,5-Dimethoxymandelic acid, di-TMS
2,5-Dimethoxyphenol, TMS
2',5'-Dimethoxypropiophenone
2,5-Hexanediol, 2TMS derivative
2,6,8-Trimethyl-4-nonyl acetate
2,6-Bis(tert-butyl)phenol, TMS derivative
2,6-Dimethyldecane
2,6-di-tert-Butyl-4-(dimethylaminomethyl)phenol
2-[2-[2-Methoxyethoxy]ethoxy-1,3-dioxalane
2-Acetoxyindane
2-Acetylmethylamino-4,5,6,7-tetrahydrobenzothiazol-7-one
2-Allyl-1,4-dimethoxybenzene
2-Amino-2-deoxyhexose, 5TMS derivative
2-Amino-4-(1-ethylpropyl)-4H-benzo[h]chromene-3-carbonitrile
2-Aminobenzoxazole, 2TBDMS derivative
2-Aminomalonic acid, N,O,O,-TMS
2-Aminosuccinonitrile
2-Benzothiazolamine, N-ethyl-
2-Butene-1,4-diol, (E)-, 2TMS derivative
2-Butylamine, N-tetradecyl-
2-Decanol, TMS derivative
2-Decene, 7-methyl-, (Z)-
2-Dodecene, (Z)-
2-Ethoxyethanol, TMS derivative
2-Ethyl-acridone
2-Ethylhexanal ethylene glycol acetal
2-Ethylhexanoic acid, TMS derivative
2H-Cyclopropa[a]naphthalen-2-one, 1,1a,4,5,6,7,7a,7b-octahydro-1,1,7,7a-tetramethyl-, (1a?,7?,7a?,7b?)-
2-Heptanol, TBDMS derivative

| |
|---|
| 2-Hexene, 3,5,5-trimethyl- |
| 2-Hexene, 3,5-dimethyl- |
| 2-Hexyl-1-octanol |
| 2-Hydroxy-2-methylbutyric acid, 2TMS derivative |
| 2-Hydroxy-4-methoxybenzaldehyde, TMS derivative |
| 2-Hydroxy-5-methoxybenzaldehyde, TMS derivative |
| 2-Hydroxymandelic acid, ethyl ester, di-TMS |
| 2-Imidazolidinone |
| 2-Isopropyl-5-methyl-1-heptanol |
| 2-Methoxybenzoic acid, 2,3-dichlorophenyl ester |
| 2-Methyl-1,2-bis(trimethylsilyloxy)butane |
| 2-Methyl-1,3-butanediol, 2TMS derivative |
| 2-Methyl-1,3-butanediol, TBDMS derivative |
| 2-Methyl-3-buten-2-ol, TMS derivative |
| 2-Methylbicyclo[3.2.1]octane |
| 2-Methyl-E-7-hexadecene |
| 2-Monomyristin, 2TMS derivative |
| 2-Monostearin, 2TMS derivative |
| 2-Naphthalenol, 8-amino- |
| 2-Nonenal |
| 2-Oxopentanoic acid, TMS derivative |
| 2-Palmitoylglycerol, 2TMS derivative |
| 2-Pentanethiol, 2-methyl- |
| 2-Phenylisopropanol, TMS derivative |
| 2-Propanol, 1-[2-(2-methoxy-1-methylethoxy)-1-methylethoxy]- |
| 2-Propanol, 2-methyl- |
| 2-Propanone, 1,1,3,3-tetrabutoxy- |
| 2-tert-Butoxytetrahydrofuran |
| 2-Undecanethiol, 2-methyl- |
| 3,3,13,13-Tetraethylpentadecane |
| 3,3-Dichloropropyne |
| 3,4-Dimethoxymandelic acid, di-TMS |
| 3,4-Dimethyl-2-(3-methyl-butyryl)-benzoic acid, methyl ester |
| 3,5-Diacetyl-4-methyl-1-phenyl-1,4-dihydropyridine |
| 3,5-Dimethoxymandelic acid, di-TMS |
| 3,5-Dimethoxymandelic amide, di-TMS |
| 3,7-Dimethyloctyl acetate |
| 3-[1-(4-Cyano-1,2,3,4-tetrahydronaphthyl)]propanenitrile |
| 3-[3-(4-Hydroxyphenyl)-3-oxoprop-1-en-1-yl]-6-methylchromen-4-one |
| 3-Allyl-5-(1H-indol-3-ylmethyl)-2-thioxo-imidazolidin-4-one |
| 3-Chlorobenzoic acid, TMS derivative |
| 3-Dodecanol, 3,7,11-trimethyl- |
| 3-Ethyl-2-methyl-2-heptanol |
| 3-Ethyl-3-methylheptane |
| 3-Ethyl-3-methylnonadecane |
| 3-Ethylpentan-3-ol, trimethylsilyl ether |
| 3H-1,2,4-Triazole-3-thione, 5-amino-1,2-dihydro- |
| 3-Hydroxy-3-phenylbutan-2-one |
| 3-Hydroxybutyric acid, 2TMS derivative |
| 3-Methoxyamphetamine, 4-trimethylsilyloxy, N-trimethylsilyl- |
| 3-Methoxy-benzoic acid 3,4-dimethyl-phenyl ester |
| 3-Methyl-1,3-bis(trimethylsilyloxy)butane |
| 3-Methyl-2-butanol, TMS derivative |
| 3-Methylvaleric acid, TMS |
| 3-pentulosonic acid, 2,4,5-tris-O-(trimethylsilyl)-, trimethylsilyl ester |
| 3-Phenanthrenol, 4b,5,6,7,8,8a,9,10-octahydro-2,4b,8,8-tetramethyl-, (4bS,8aS)- |
| 3-Phenyl-2-butanol, TMS derivative |
| 3-Phenyllactic acid, 2TMS derivative |

| |
|---|
| 3-Pyridinol, TMS derivative |
| 4b,8-Dimethyl-2-isopropylphenanthrene, 4b,5,6,7,8,8a,9,10-octahydro- |
| 4-Benzyl-4,5-dihydroisoxazole |
| 4-Chlorobenzoic acid, TMS derivative |
| 4-Cyano-4-methylthio-tetracyclo(6,2,1,1(3,6).0(2,7)dodec-9-ene |
| 4-Decene, 7-methyl-, (E)- |
| 4-Dodecene, (E)- |
| 4-Hydroxy-3,4,6-trimethylhept-5-enoic acid lactone |
| 4-Methoxybenzoic acid, 4-isopropylphenyl ester |
| 4-Methoxyphenol, TMS derivative |
| 4-Methylethcathinone, N-trimethylsilyl- |
| 4-Methylpentan-2-ol, tert-butyldimethylsilyl ether |
| 4-Methylvaleric acid, TMS derivative |
| 4-Pentene-2-ol, 2-methyl |
| 4-Pyridinol, TMS derivative |
| 4-Trimethylsilylphenol |
| 5,2,1,6,3,4-[2,3]Butanediyl[1,4]diylidenedipentaleno[2,1,6-cde:2',1',6'-gha]pentalene, hexadecahydro- |
| 5,5-Dimethyl-1,3-dioxane-2-ethanol, TMS derivative |
| 5,6,7,8,9,10-Hexahydrobenzocyclooctene |
| 5,6-Dimethyl-4-phenyl-3-cyanopyridine-2(1H)-thione |
| 5?-Androstan-3?-ol, O-acetyl- |
| 5?-Androstan-3?-ol, O-ethyl- |
| 5-Cyano-2-methyl-4-methylthio-6-phenylpyrimidine |
| 5-Dodecene, (E)- |
| 5-Eicosene, (E)- |
| 5-Ethyldecane |
| 5-Hydroxytryptophan, 4TMS derivative |
| 5-Methyl-2-(2-methyl-2-tetrahydrofuryl)tetrahydrofuran |
| 5-Methyluridine, 3TMS derivative |
| 5-Nitro-3-phenyl-1H-indazole |
| 5-Nonanol, TMS derivative |
| 5-Oxoproline, 2TMS derivative |
| 6-(4-Fluorophenyl)-4-phenyl-3-cyanopyridine-2(1H)-thione |
| 6,9-Heptadecadiene |
| 6-Dodecyne |
| 6-Methoxy-3-methylbenzofuran |
| 6-Nonenal, (E)- |
| 6-Tetradecene, (E)- |
| 6-Tridecene |
| 7,9-Di-tert-butyl-1-oxaspiro(4,5)deca-6,9-diene-2,8-dione |
| 7-Acetyl-6-ethyl-1,1,4,4-tetramethyltetralin |
| 7-Isopropyl-1,1,4a-trimethyl-1,2,3,4,4a,9,10,10a-octahydrophenanthrene |
| 7-Methyl-Z-tetradecen-1-ol acetate |
| 7-Tetradecene, (E)- |
| 8-Hexadecyne |
| 8-Methyl-6-nonenamide |
| 8-Methylnonanoic acid, trimethylsilyl ester |
| 9(E),11(E)-Conjugated linoleic acid, trimethylsilyl ester |
| 9,10-Anthracenedione, 1,4-diamino-2,3-dihydro- |
| 9,11-Octadecadienoic acid, methyl ester, (E,E)- |
| 9,12,15-Octadecatrienoic acid, ethyl ester, (Z,Z,Z)- |
| 9,12-Octadecadienoic acid (Z,Z)- |
| 9,12-Octadecadienoic acid (Z,Z)-, 2-hydroxy-1-(hydroxymethyl)ethyl ester |
| 9,12-Octadecadienoic acid (Z,Z)-, methyl ester |
| 9,12-Octadecadienoic acid, ethyl ester |
| 9,12-Octadecadienoic acid, methyl ester |
| 9-Octadecen-1-ol, (Z)- |
| 9-Octadecen-1-ol, acetate, (Z)- |

| |
|---|
| 9-Octadecenamide, (Z)- |
| 9-Octadecenenitrile, (Z)- |
| 9-Octadecenoic acid, (E)-, TMS derivative |
| 9-Octadecynenitrile |
| 9-Tricosene, (Z)- |
| Acetic acid, 7,7,10a,12a-tetramethyl-2,5-dioxo-1,2,3,4,4a,4b,5,7,8,9,10,10a,10b,11,12,12a-hexadecahydro-1-azachrysen-8-yl ester |
| Acetic acid, bis[(trimethylsilyl)oxyl]-, trimethylsilyl ester |
| Acetic acid, dimethoxy-, methyl ester |
| Acetic acid, TMS derivative |
| Acrylic acid, 2,3-bis[(trimethylsilyl)oxy]-, trimethylsilyl ester |
| Adonitol, 5TMS derivative |
| Ala-?-Ala, N-trimethylsilyl-, trimethylsilyl ester |
| Alanine, N-methyl-n-butoxycarbonyl-, undecyl ester |
| Alanine, N-methyl-N-ethoxycarbonyl-, dodecyl ester |
| Alanine, N-methyl-N-ethoxycarbonyl-, pentadecyl ester |
| Alanine, N-methyl-N-isobutoxycarbonyl-, isohexyl ester |
| Alanine, N-methyl-N-methoxycarbonyl-, undecyl ester |
| Alanylalanine, N,N'-dimethyl-N'-methoxycarbonyl-, hexyl ester |
| Alanylalanine, N,N'-dimethyl-N'-methoxycarbonyl-, pentyl ester |
| Alanylglycine, 2TMS derivative |
| Allonic acid, ?-lactone, 4TMS derivavative |
| alpha-(4-DIMETHYLAMINOPHENYL)-?-(9-PHENANTHRYL)DECANE |
| alpha-D-(-)-Lyxopyranose, 4TMS derivative |
| alpha-D-(+)-Talopyranose, 5TMS derivative |
| alpha-D-Allopyranose, 5TMS derivative |
| alpha-D-Arabinopyranose, 4TMS derivative |
| alpha-D-Glucopyranose, 5TMS derivative |
| alpha-D-Mannopyranose, 5TMS derivative |
| alpha-D-Xylopyranose, 4TMS derivative |
| alpha-Farnesene |
| alpha-Hydroxyisobutyric acid, 2TMS derivative |
| alpha-L-6-Deoxymannopyranose, 4TMS derivative |
| alpha-Methyl-L-tyrosine, 3TMS derivative |
| Androstane, (5?)- |
| Anthra[1,9-cd]pyrazol-6(2H)-one |
| Anthranilic acid, TMS derivative |
| Arabinitol, 5TMS derivative |
| Arabinofuranose, 1,2,3,5-tetrakis-O-(trimethylsilyl)- |
| Arabinose, 4TMS derivative |
| Arachidic acid, TMS derivative |
| Aspartic acid, 3TMS derivative |
| Behenic acid, TMS derivative |
| Benzaldehyde, 2,5-dimethoxy- |
| Benzamide, N-(1,1-dimethylethyl)- |
| Benzamide, N-propyl- |
| Benzenamine, 4-(6-methyl-2-benzothiazolyl)- |
| Benzenamine, N,N-diethyl-3-methyl- |
| Benzenamine, N,N-diethyl-4-methyl- |
| Benzene, (1-butylheptyl)- |
| Benzene, (1-methyldodecyl)- |
| Benzene, (1-methylethyl)- |
| Benzene, (1-methyltridecyl)- |
| Benzene, (1-methylundecyl)- |
| Benzene, (1-pentylheptyl)- |
| Benzene, [1-[[1-(1-methylethyl)-3-butenyl]oxy]ethyl]-, [S-(R*,R*)]- |
| Benzene, 1,1'-(1-ethenyl-1,3-propanediyl)bis- |
| Benzene, 1,1'-(oxydiethylidene)bis- |

| |
|---|
| Benzene, 1,2,3-trimethyl- |
| Benzene, 1,2,4-trimethyl- |
| Benzene, 1,4-dimethoxy- |
| Benzene, 1-ethyl-2-methyl- |
| Benzene, 1-ethyl-3-methyl- |
| Benzene, 1-ethyl-4-methyl- |
| Benzene, 1-methyl-4-propyl- |
| Benzene, 4-butyl-1,2-dimethoxy- |
| Benzene, propyl- |
| Benzeneacetic acid, TBDMS derivative |
| Benzeneethanol, ?-ethenyl- |
| Benzenemethanol, ?-(1-aminobutyl)-, N-trimethylsilyl-, trimethylsilyl ether |
| Benzenemethanol, ?,?-dimethyl- |
| Benzenepropanoic acid, 3,5-bis(1,1-dimethylethyl)-4-hydroxy-, methyl ester |
| Benzoic acid, 2-hydroxy-3-[(2-hydroxy-4-methoxy-6-propylbenzoyl)oxy]-4-methoxy-6-propyl-, methyl ester |
| Benzoic acid, methyl ester |
| Benzoic Acid, TBDMS derivative |
| Benzoic Acid, TMS derivative |
| Benzophenone |
| Benzothiazole |
| Benzothiazole, 2-(4-amino-3-methylphenyl)- |
| Benzothiazole, 2-(o-aminophenyl)-4-methyl- |
| Benzyl alcohol, TMS derivative |
| beta-D-(+)-Mannopyranose, 5TMS derivative |
| beta-D-(+)-Talopyranose, 5TMS derivative |
| beta-D-Allopyranose, 5TMS derivative |
| beta-D-Glucopyranose, 5TMS derivative |
| beta-Hydroxypyruvic acid, trimethylsilyl ether, trimethylsilyl ester |
| beta-L-Arabinopyranose, 4TMS derivative |
| Biphenyl-4,4'-dicarboxylic acid, bis-(4-pentyl-phenyl ester |
| Bisphenol A monomethyl ether, TMS derivative |
| Bisphenol A, 2TMS derivative |
| Bisphenol A, TMS derivative |
| Boric acid, 3TMS derivative |
| Butanal, oxime |
| Butane, 2,3-dimethoxy-2-methyl- |
| Butane-1,3-diol, 1-methylene-3-methyl-, bis(trimethylsilyl)ether |
| Butanedioic acid, 2TMS derivative |
| Butyl 9,12,15-octadecatrienoate |
| Butyl 9,12-octadecadienoate |
| Butylated Hydroxytoluene |
| Butylone, N-trimethylsilyl- |
| Butyric Acid, TMS derivative |
| Cadaverine, 4TMS derivative |
| Carbitol, TMS derivative |
| Chavicol TMS |
| Cholesterol, TMS derivative |
| Chrysophanol, 2TMS derivative |
| Citric acid, 4TMS derivative |
| Cyanuric acid, 3TMS derivative |
| Cyclobutanecarboxylic acid, 2-propenyl ester |
| Cyclododecane |
| Cyclohexane, (1-hexadecylheptadecyl)- |
| Cyclohexane, 1-methyl-4-(1-methylethenyl)-, trans- |
| Cyclohexanemethanol, 4-methyl-, trans- |
| Cyclohexanol, TMS derivative |
| Cyclohexanone, 3,3,5-trimethyl- |
| Cyclohexene, 1-butyl- |

| |
|---|
| Cyclohexene,3-propyl- |
| Cyclooctane, 1,4-dimethyl-, cis- |
| Cyclopenta[g]-2-benzopyran, 1,3,4,6,7,8-hexahydro-4,6,6,7,8,8-hexamethyl- |
| Cyclopentane, 1,1,3,3-tetramethyl- |
| Cyclopentane, 1,1'-hexadecylidenebis- |
| Cyclopentane, nonyl- |
| Cyclopentane, undecyl- |
| Cyclopentanone, 2,2,4-trimethyl- |
| Cyclopropane, 1-(1-hydroxy-1-heptyl)-2-methylene-3-pentyl- |
| Cyclopropane, 1-hexyl-2-methyl- |
| Cyclopropane, 1-methyl-2-(3-methylpentyl)- |
| D-(-)- Erythrofuranose, tris(trimethylsilyl) ether (isomer 1) |
| D-(-)-Fructofuranose, pentakis(trimethylsilyl) ether (isomer 1) |
| D-(-)-Fructofuranose, pentakis(trimethylsilyl) ether (isomer 2) |
| D-(-)-Fructopyranose, 5TMS derivative (isomer 1) |
| D-(-)-Ribofuranose, tetrakis(trimethylsilyl) ether (isomer 1) |
| D-(-)-Ribofuranose, tetrakis(trimethylsilyl) ether (isomer 2) |
| D-(-)-Tagatofuranose, pentakis(trimethylsilyl) ether (isomer 1) |
| D-(-)-Tagatofuranose, pentakis(trimethylsilyl) ether (isomer 2) |
| D-(+)-Arabitol, 5TMS derivative |
| D-(+)-Galactopyranose, 5TMS derivative (isomer 2) |
| D-(+)-Talofuranose, pentakis(trimethylsilyl) ether (isomer 2) |
| D-(+)-Xylose, 4TMS derivative |
| D-Alanine, N-ethoxycarbonyl-, undecyl ester |
| D-Allofuranose, pentakis(trimethylsilyl) ether |
| D-Arabinopyranose, 4TMS derivative (isomer 2) |
| Decane |
| Decane, 2,3,5-trimethyl- |
| Decane, 2,6,7-trimethyl- |
| Decane, 4-methyl- |
| Decane, 5,6-dimethyl- |
| Decanoic acid, TMS derivative |
| Decyl oleate |
| D-Erythro-Pentitol, 2-deoxy-1,3,4,5-tetrakis-O-(trimethylsilyl)- |
| D-Fructose, 5TMS derivative |
| D-Fucitol, 5TMS derivative |
| D-Glucopyranose, 5TMS derivative |
| D-Glucose, 5TMS derivative |
| Dicyclopentadiene |
| Diethyl aminomalonate, TMS derivative |
| Diethylene glyco, TMS derivative |
| Diethylene glycol, 2TMS derivative |
| Diethylene glycol, n-butyl ether, trimethylsilyl ether |
| Diglycerol, 4TMS derivative |
| Dihydroxyacetone, 2TMS derivative |
| Dimethyl hexopyranosiduronate, ?-D-, 3TMS derivative |
| Dinaphtho(1,2-b:2',1'-d)thiophene |
| Disulfide, di-tert-dodecyl |
| dl-2-Phenyl-1,2-propanediol |
| dl-7-Azatryptophan |
| dl-Alanine ethyl ester |
| DL-Arabinopyranose, 4TMS derivative |
| D-Lyxose, 4TMS derivative |
| D-Mannopyranose, 5TMS derivative |
| D-Mannose, 5TMS derivative |
| D-Norleucine, N-ethoxycarbonyl-, dodecyl ester |
| Docosane |
| Docosanol, TBDMS derivative |

| |
|---|
| Docosyl octyl ether |
| Dodecane |
| Dodecane, 2,6,11-trimethyl- |
| Dodecane, 2,7,10-trimethyl- |
| Dodecane, 4,6-dimethyl- |
| Dodecanoic acid, ethenyl ester |
| Dodecanoic acid, methyl ester |
| Dodecanoic acid, TMS derivative |
| Dopamine, 4TMS derivative |
| D-Psicofuranose, pentakis(trimethylsilyl) ether (isomer 1) |
| D-Psicofuranose, pentakis(trimethylsilyl) ether (isomer 2) |
| D-Psicopyranose, 5TMS derivative (isomer 2) |
| D-Ribofuranose, 5-deoxy-5-(methylsulfinyl)-1,2,3-tris-O-(trimethylsilyl)- |
| D-Ribose, 4TMS derivative |
| D-Xylopyranose, 4TMS derivative |
| D-Xylose, 4TMS derivative |
| E-11(12-Cyclopropyl)dodecen-1-ol acetate |
| E-11-Hexadecen-1-ol |
| E-8-Methyl-9-tetradecen-1-ol acetate |
| E-9-Tetradecenoic acid |
| Eicosane |
| Eicosane, 2-methyl- |
| Eicosanenitrile |
| Eicosanoic acid, methyl ester |
| Eicosyl nonyl ether |
| Ephedrine, 2TMS derivative |
| Erythritol, 4TMS derivative |
| Ethanimidic acid, N-(trimethylsilyl)-, trimethylsilyl ester |
| Ethanone, 1,1'-(1,4-phenylene)bis- |
| Ethyl 3-hydroxybutyrate, TMS derivative |
| Ethyl 9,12-hexadecadienoate |
| Ethylene glycol - Adipate - Diethylene glycol |
| Ethylene glycol butyl ether, trimethylsilyl ether |
| Ethylene glycol, 2TMS derivative |
| Ethylene glycol, TMS derivative |
| Ethylene sebacate |
| Formamide, N-methyl- |
| Fructofuranoside, methyl 1,3,4,6-tetrakis-O-(trimethylsilyl)-, ?-D- |
| Fumaric acid, pent-4-en-2-yl tetradecyl ester |
| Furfuryl alcohol, TMS derivative |
| Galactopyranose, 5TMS derivative |
| Gluconolactone (3R,4S,5R,6R)-, 4TMS derivative |
| Gluconolactone, 4TMS derivative |
| Glucopyranose, 5TMS derivative |
| Glucose, 5TMS derivative |
| Glucuronolactone, trisO-(trimethylsilyl)- |
| Glyceric acid, 3TMS derivative |
| Glycerol monostearate, 2TMS derivative |
| Glycerol, 3TMS derivative |
| Glycine, 3TMS derivative |
| Glycine, di-TMS |
| Glycine, N-methyl-N-ethoxycarbonyl-, decyl ester |
| Glycine, N-methyl-N-ethoxycarbonyl-, nonyl ester |
| Glycine, N-methyl-N-ethoxycarbonyl-, octyl ester |
| Glycolic acid, 2TMS derivative |
| Glyoxime, 2TMS derivative |
| Heneicosane |
| Heneicosane, 11-decyl- |

| |
|---|
| Heneicosane, 3-methyl- |
| Heneicosanoic acid, TMS derivative |
| Henicos-1-ene |
| Hentriacontane |
| Heptacosane |
| Heptadecane |
| Heptadecane, 2,6,10,15-tetramethyl- |
| Heptadecane, 2-methyl- |
| Heptadecanenitrile |
| Heptadecanoic acid, glycerine-(1)-monoester, bis-O-trimethylsilyl- |
| Heptadecanoic acid, TMS derivative |
| Heptamethyl-3-phenyl-1,4-cyclohexadiene |
| Heptanamide, 4-ethyl-5-methyl- |
| Heptane, 2,2,4,6,6-pentamethyl- |
| Heptane, 2,5,5-trimethyl- |
| Heptane, 3,3,5-trimethyl- |
| Heptanoic acid, TMS derivative |
| Hexacosane |
| Hexacosanoic acid, TMS derivative |
| Hexadecanamide |
| Hexadecane |
| Hexadecane, 2,6,10,14-tetramethyl- |
| Hexadecane, 2,6,11,15-tetramethyl- |
| Hexadecane, 2-methyl- |
| Hexadecane, 7-methyl- |
| Hexadecane-1,2-diol |
| Hexadecanenitrile |
| Hexadecanoic acid, butyl ester |
| Hexadecanoic acid, methyl ester |
| Hexan-3-ol, trimethylsilyl ether |
| Hexane, 4-ethyl-2-methyl- |
| Hexanedioic acid, 2TMS derivative |
| Hexanedioic acid, bis(2-ethylhexyl) ester |
| Hexanedioic acid, dioctyl ester |
| Hexanoic acid, TMS derivative |
| Hexatriacontane |
| Hexopyranose, 5TMS derivative |
| Hexyl octyl ether |
| Hydroquinone, TMS derivative |
| Hydroxylamine, methyl-(1-phenylethyl)- |
| Hydroxylamine, O-(3-methylbutyl)- |
| Hydroxylamine, O-pentyl- |
| Indane |
| Indeno[2',1':4,5]thieno[3,2-b]thiopyran |
| Inosose-2, 1,3,4,5,6-pentakis-O-(trimethylsilyl)-, myo- |
| Isobutanol, TMS derivative |
| Isobutyl triacontyl ether |
| Isopropyl alcohol, TMS derivative |
| Isopropyl palmitate |
| Isopropylamine, N-tert-butyldimethylsilyl- |
| Isoquinoline, 1,2,3,4-tetrahydro-6,7-dimethoxy- |
| Isoquinoline, 3,4-dihydro-6,7-dimethoxy-1-[3-methoxyphenyl]- |
| L-(-)-Arabitol, 5TMS derivative |
| L-(-)-Sorbofuranose, pentakis(trimethylsilyl) ether |
| L-(+)-Rhamnopyranose, 4TMS derivative |
| L-5-Oxoproline, , 2TMS derivative |
| Labda-8(20),14-dien-13-ol, (13S)-, O-TMS |
| Lactic acid dimer, bis(trimethylsilyl)- |

| |
|---|
| Lactic Acid, 2TMS derivative |
| l-Alanine ethylamide, (S)- |
| L-Alanine, 2TMS derivative |
| L-Aspartic acid, diethyl ester |
| Levoglucosan, 3TMS derivative |
| Levulinic acid, TMS derivative |
| L-Homophenylalanine, N-(n-pentyl)-, pentyl ester |
| Lignoceric acid, TMS derivative |
| Linoleyl acetate |
| Linolool oxide, (Z)-, TMS derivative |
| l-Norleucine, N-ethoxycarbonyl-, decyl ester |
| l-Norleucine, N-methoxycarbonyl-, isohexyl ester |
| l-Norleucine, N-methoxycarbonyl-, octyl ester |
| l-Norleucine, N-methoxycarbonyl-, pentyl ester |
| l-Norvaline, N-ethoxycarbonyl-, isohexyl ester |
| l-Norvaline, N-ethoxycarbonyl-, nonyl ester |
| l-Norvaline, N-methoxycarbonyl-, heptyl ester |
| l-Norvaline, N-methoxycarbonyl-, nonyl ester |
| l-Norvaline, N-methoxycarbonyl-, octyl ester |
| l-Norvaline, N-methoxycarbonyl-, pentyl ester |
| l-Norvaline, N-methoxycarbonyl-, tetradecyl ester |
| l-Norvaline, n-propargyloxycarbonyl-, heptyl ester |
| l-Norvaline, n-propargyloxycarbonyl-, propargyl ester |
| l-Norvaline, n-propoxycarbonyl-, undecyl ester |
| L-Norvaline, trimethylsilyl ester |
| L-Phenylalanine, 2TMS derivative |
| L-Rhamnose, 4TMS derivative |
| L-Sorbopyranose, (1S,2R,3S)-, 5TMS derivative |
| L-Threitol, 4TMS derivative |
| L-Threonine, 3TMS derivative |
| L-Val-L-Leu, N-trimethylsilyl-, trimethylsilyl ester |
| Malic acid, 3TMS derivative |
| Malonic acid, bis(2-trimethylsilylethyl ester |
| m-Aminophenylacetylene |
| Mercaptoethanol, 2TMS derivative |
| Mesitylene |
| meso-Erythritol, 4TMS derivative |
| Metanephrine, 3TMS derivative |
| Methamphetamine, N-TMS |
| Methiopropamine, N-trimethylsilyl- |
| Methyl (2R,3R,4S)-2,4-dimethyl-3-hydroxyhexanoate |
| Methyl (3,4-dimethoxyphenyl)(hydroxy)acetate, TMS derivative |
| Methyl 4-methoxysalicylate, TMS derivative |
| Methyl 9-cis,11-trans-octadecadienoate |
| Methyl galactoside, 4TMS derivative |
| Methyl pentopyranoside, 3TMS derivative |
| Methyl stearate |
| Methyl vinyl ketone |
| Methyl Z-11-tetradecenoate |
| Monolaurin, 2TMS derivative |
| m-Toluic acid, TMS derivative |
| Myristic acid, TMS derivative |
| Naphthalene |
| Naphthalene, 1,2,3,4-tetrahydro-1,6-dimethyl-4-(1-methylethyl)-, (1S-cis)- |
| Naphthalene, 1-methyl- |
| Naphthalene, 5-ethyl-1,2,3,4-tetrahydro- |
| Naphthalene, 6-ethyl-1,2,3,4-tetrahydro- |
| n-Hexadecanoic acid |

| |
|---|
| n-Nonadecanol-1 |
| Nonacosan-10-ol, O-TMS |
| Nonadecanenitrile |
| Nonadecanoic acid, TMS derivative |
| Nonane, 2,6-dimethyl- |
| Nonane, 5-methyl-5-propyl- |
| Nonanoic acid, TMS derivative |
| n-Propyl 9,12-octadecadienoate |
| N-Trimethylsilylstearamide |
| Octacosane |
| Octadecanamide |
| Octadecanamide, N-(2-methylpropyl)-N-nitroso- |
| Octadecane |
| Octadecane, 2-methyl- |
| Octadecanenitrile |
| Octadecanoic acid, 2-methylpropyl ester |
| Octadecanoic acid, 2-oxo-, methyl ester |
| Octadecanoic acid, ethenyl ester |
| Octadecanoic acid, phenylmethyl ester |
| Octaethylene glycol, 2TMS derivative |
| Octan-2-yl palmitate |
| Octane, 2,3,6,7-tetramethyl- |
| Octane, 2,3,7-trimethyl- |
| Octane, 3,3-dimethyl- |
| Octane, 3,5-dimethyl- |
| Octane, 6-ethyl-2-methyl- |
| Octanoic acid, TMS derivative |
| Octopamine, 4TMS derivative |
| Oleamide, TMS derivative |
| Oleic Acid |
| Oleic Acid, (Z)-, TMS derivative |
| Oleic acid, butyl ester |
| Orthoformic acid, triisobutyl ester |
| Oxalic acid, 2TMS derivative |
| Oxalic acid, heptyl propyl ester |
| Oxime-, methoxy-phenyl-_ |
| Palmitelaidic acid, TMS derivative |
| Palmitic Acid, TMS derivative |
| Palmitoleamide |
| Palmitoleonitrile |
| p-Anisic acid, tridec-2-ynyl ester |
| Pentacosane |
| Pentacosanoic acid, trimethylsilyl ester |
| Pentadecane |
| Pentadecane, 2,6,10,14-tetramethyl- |
| Pentadecane, 2,6,10-trimethyl- |
| Pentadecanoic acid, 14-methyl-, methyl ester |
| Pentadecanoic acid, glycerine-(1)-monoester, bis-O-trimethylsilyl- |
| Pentadecanoic acid, TMS derivative |
| Pentanamide |
| Pentanoic acid, 2,4-dimethyl-3-oxo-, methyl ester |
| Pentanoic acid, 2-[(trimethylsilyl)oxy]-, trimethylsilyl ester |
| Pentanoic acid, 3-methyl-2-oxo-, methyl ester |
| Pentanoic acid, 4-oxo-, pentyl ester |
| Pentyl linoleate |
| Phenanthrene, 1,2,3,4,4a,9,10,10a-octahydro-1,1,4a-trimethyl-7-(1-methylethyl)-, (4aS-trans)- |
| Phenethylamine, ?-methyl-N-propyl- |
| Phenol, 2,4-bis(1-methylethyl)-, acetate |

| |
|---|
| Phenol, 2,6-bis(1,1-dimethylethyl)-4-methyl-, methylcarbamate |
| Phenol, 4,6-di(1,1-dimethylethyl)-2-methyl- |
| Phenol, TMS derivative |
| phenoxyethanol, TMS derivative |
| p-Hydroxydiisopropylbenzene, TMS derivative |
| Pipecolic acid, 2TMS derivative |
| Pipecolic acid, N-ethoxycarbonyl-, dodecyl ester |
| Pipecolic acid, N-octyloxycarbonyl-, undecyl ester |
| Propanal, 2-methyl- |
| Propanamide, N-(1-naphthyl)-2-methyl- |
| Propanedinitrile, cyclohexyl(2-methylcyclohexyl)- |
| Propanetriol, 2-methyl-, tris-O-(trimethylsilyl)- |
| Propanoic acid, 2-methyl-, hexyl ester |
| Propylene glycol, 2TMS derivative |
| Propylparaben, TMS derivative |
| Pyrogallol, 3TMS derivative |
| Pyroglutamic acid, TMS derivative |
| Pyrrole-2-carboxylic acid, 2TMS derivative |
| Pyruvic acid, butyl ester |
| Pyruvic acid, TMS derivative |
| Quinoline, 1,2-dihydro-2,2,4-trimethyl- |
| Resorcinol, TMS derivative |
| Ribitol, 5TMS derivative |
| Ribonic acid, 2,3,4,5-tetrakis-O-(trimethylsilyl)-, trimethylsilyl ester |
| Salsoline |
| Serine, 3TMS derivative |
| s-Indacen-1(2H)-one, 3,5,6,7-tetrahydro-3,3,4,5,5,8-hexamethyl- |
| Squalene |
| Stearic acid, TMS derivative |
| Sulfurous acid, isobutyl pentyl ester |
| Supraene |
| Synephrine, N,O,O'-tris-trimethylsilyl |
| Talose, 5TMS derivative |
| Tetracosanol, O-TMS |
| Tetracyano-p-quinodimethane |
| Tetradecane |
| Tetradecanenitrile |
| Tetradecanoic acid, 10,13-dimethyl-, methyl ester |
| Tetradecanoic acid, 2-oxo-, ethyl ester |
| Tetradecanoic acid, dimethyl(isopropyl)silyl ester |
| Tetrahydrogeranyl formate |
| Thiourea, tetramethyl- |
| Tolycaine |
| trans,trans-9,12-Octadecadienoic acid, propyl ester |
| trans-13-Octadecenoic acid |
| trans-9-Octadecenoic acid, pentyl ester |
| Tri(1,2-propyleneglycol), monomethyl ether |
| Tributyl acetylcitrate |
| Tridecane |
| Tridecane, 3-methylene- |
| Tridecane, 4-methyl- |
| Tridecanoic acid, TMS derivative |
| Triethanolamine, 3TMS derivative |
| Triethylene glycol, 2TMS derivative |
| Trimethylolpropane, 3TMS derivative |
| Trimethylsilyl tricosanoate |
| Trimethylsilylserine |
| Triphenylphosphine oxide |

| |
|---|
| Tris(trimethylsilyl)carbamate |
| Trisaminol, 3-OTMS |
| Tromethamine, 4TMS derivative |
| Undecane, 2,5-dimethyl- |
| Undecane, 2,6-dimethyl- |
| Undecane, 3,9-dimethyl- |
| Undecane, 3-methyl- |
| Undecane, 4-methyl- |
| Undecane, 5-methyl- |
| Undecanoic acid, 11-amino- |
| Uracil, 2TMS derivative |
| Urazole |
| Urea, 2TMS derivative |
| Valine, N-methyl-N-ethoxycarbonyl-, isohexyl ester |
| Xylitol, 5TMS derivative |
| Xylose, 4TMS derivative |
| Z-(13,14-Epoxy)tetradec-11-en-1-ol acetate |

Table S2: Molecules with different abundances between apple groups

| | FC | raw.pval |
|---|---|---|
| 1,2,3-Butanetriol, 3TMS derivative | 43.391 | 0.001848 |
| 1,2-Benzenedicarboxylic acid, bis2-ethylhexyl ester | 3.1702 | 0.016918 |
| 1,2-Benzenediol, O-4-methoxybenzoyl -O'-2-furoyl- | 2.7326 | 0.003042 |
| 1,2-Cyclohexanedicarboxylic acid, heptadecyl 2-methylcyclohexyl ester | 2.2178 | 0.019562 |
| 1-Butoxy-2-propanol, TMS derivative | 2.5963 | 0.008612 |
| 1-Chloroundecane | 3.1714 | 0.00242 |
| 1-Ethoxycarbonylmethylpyridinium bromide | 1.7768 | 0.000777 |
| 1H-Pyrazolo3,4-dpyrimidin-4-amine | 0.57424 | 0.013792 |
| 1H-Quinolin-2-one, 5,8-dimethoxy-1,4-dimethyl- | 0.55028 | 0.033273 |
| 1-Methyl-5-mercaptotetrazole | 0.50372 | 0.047957 |
| 1-Octacosanol, 2,4,6,8-tetramethyl-, all-R- | 0.53936 | 0.002067 |
| 1-Tetradecene | 2.3593 | 0.018466 |
| 1-Undecanol, TMS derivative | 1.6669 | 0.029171 |
| 2,4,6-Tris1,1-dimethylethyl-4-methylcyclohexa-2,5-dien-1-one | 2.582 | 0.035173 |
| 2,4-Dichlorobenzoic acid, TMS derivative | 9.7553 | 4.44E-06 |
| 2,5-Cyclohexadiene-1,4-dione, 2,6-bis1,1-dimethylethyl- | 0.65213 | 0.005792 |
| 2,5-Dimethoxyphenylacetone | 2.9865 | 0.027463 |
| 2,6,8-Trimethyl-4-nonyl acetate | 1.6622 | 0.004345 |
| 2,6-Dimethyldecane | 2.1435 | 0.026926 |
| 2,6-di-tert-Butyl-4-dimethylaminomethylphenol | 1.7458 | 0.009767 |
| 2-Acetoxyindane | 0.47058 | 0.014991 |
| 2-Amino-2-deoxyhexose, 5TMS derivative | 0.32526 | 0.006777 |
| 2-Benzothiazolamine, N-ethyl- | 0.63035 | 0.01005 |
| 2-Butylamine, N-tetradecyl- | 2.6107 | 1.76E-06 |
| 2-Ethoxyethanol, TMS derivative | 3.2334 | 0.046455 |
| 2-Propanol, 1-2-2-methoxy-1-methylethoxy-1-methylethoxy- | 7.2175 | 0.013011 |
| 2-Propanol, 2-methyl- | 2.1136 | 0.02089 |
| 3,4-Dimethoxymandelic acid, di-TMS | 0.41598 | 0.000826 |
| 3,5-Diacetyl-4-methyl-1-phenyl-1,4-dihydropyridine | 0.61988 | 0.004208 |
| 3,5-Dimethoxymandelic acid, di-TMS | 0.55152 | 0.04622 |
| 3,5-Dimethoxymandelic amide, di-TMS | 3.0032 | 0.008269 |
| 3-Chlorobenzoic acid, TMS derivative | 7.528 | 4.79E-05 |
| 3-Ethyl-2-methyl-2-heptanol | 2.4836 | 0.025418 |
| 3-Ethyl-3-methylheptane | 1.8323 | 0.002771 |

| | | |
|---|---|---|
| 3H-1,2,4-Triazole-3-thione, 5-amino-1,2-dihydro- | 0.47736 | 0.016553 |
| 3-Methoxy-benzoic acid 3,4-dimethyl-phenyl ester | 2.2539 | 0.017459 |
| 3-Methyl-2-butanol, TMS derivative | 1.6862 | 0.024834 |
| 4,4-Dimethyl-5-methylene-4,5-dihydro-3H-pyrrol-2-yl-4,4-dimethyl-5-methylene-pyrrolidin-2-ylidene-acetonitrile | 0.61988 | 0.004208 |
| 4-Chlorobenzoic acid, TMS derivative | 7.5359 | 4.77E-05 |
| 4-Decene, 7-methyl-, E- | 1.7794 | 0.008838 |
| 4-Methoxybenzoic acid, 4-isopropylphenyl ester | 1.8093 | 0.012482 |
| 5,2,1,6,3,4-2,3Butanediyl1,4diylidenedipentaleno2,1,6-cde2',1',6'-ghapentalene, hexadecahydro- | 0.44444 | 0.002067 |
| 5-Phenylvaleric acid, tert-butyldimethylsilyl ester | 3.347 | 0.00341 |
| 6-Methoxy-3-methylbenzofuran | 14.175 | 0.000413 |
| 9,12-Octadecadienoic acid Z,Z-, methyl ester | 3.0499 | 0.000197 |
| 9E,11E-Conjugated linoleic acid, trimethylsilyl ester | 1.669 | 0.001769 |
| 9-Octadecenoic acid, E-, TMS derivative | 1.6862 | 0.024834 |
| Alanylalanine, N,N'-dimethyl-N'-methoxycarbonyl-, hexyl ester | 0.48236 | 0.017379 |
| alpha-Farnesene | 4.6891 | 0.000584 |
| Anthra1,9-cdpyrazol-62H-one | 6.8279 | 0.020787 |
| Anthranilic acid, TMS derivative | 5.0707 | 0.000411 |
| Benzaldehyde, 2,5-dimethoxy- | 0.4192 | 0.000115 |
| Benzaldehyde, 2-hydroxy-5-nitro-, 2-iodophenylhydrazone | 1.8687 | 0.003163 |
| Benzenamine, 4-6-methyl-2-benzothiazolyl- | 3.8256 | 1.34E-08 |
| Benzene, 1,1'-1-ethenyl-1,3-propanediylbis- | 0.54504 | 0.01181 |
| Benzene, 1-pentylheptyl- | 2.1272 | 0.005165 |
| Benzeneacetic acid, TMS derivative | 5.3755 | 0.007656 |
| Benzothiazole, 2-o-aminophenyl-4-methyl- | 2.2101 | 6.35E-05 |
| Boric acid, 3TMS derivative | 0.29856 | 0.00099 |
| Butanal, oxime | 2.3494 | 0.007812 |
| Chrysophanol, 2TMS derivative | 1.7043 | 0.006695 |
| Cyclopentane, 1,1'-hexadecylidenebis- | 0.4522 | 0.005536 |
| Cyclopropane, 1-1-hydroxy-1-heptyl-2-methylene-3-pentyl- | 0.23274 | 7.72E-05 |
| Dinaphtho1,2-b2',1'-dthiophene | 2.3156 | 0.018455 |
| Docosanol, TBDMS derivative | 1.9921 | 0.024672 |
| Dodecane, 4,6-dimethyl- | 1.5406 | 0.007414 |
| D-Xylopyranose, 4TMS derivative | 1.9769 | 0.044205 |
| Eicosane | 2.3172 | 0.037552 |
| Ethanimidic acid, N-trimethylsilyl-, trimethylsilyl ester | 0.38458 | 0.047868 |
| Formamide, N-methyl- | 2.4858 | 0.024985 |
| Glycerol monostearate, 2TMS derivative | 5.0057 | 0.010721 |
| Glycine, N-methyl-N-ethoxycarbonyl-, nonyl ester | 0.48215 | 0.021337 |
| Heneicosane, 3-methyl- | 7.2758 | 0.000941 |
| Heptane, 2,2,4,6,6-pentamethyl- | 2.1414 | 0.014204 |
| Hexadecanamide | 3.1738 | 0.002398 |
| Indeno2',1'4,5thieno3,2-bthiopyran | 3.6723 | 1.51E-05 |
| Inosose-2, 1,3,4,5,6-pentakis-O-trimethylsilyl-, myo- | 0.51892 | 0.039516 |
| Isopropylamine, N-tert-butyldimethylsilyl- | 0.47798 | 0.01671 |
| Labda-820,14-dien-13-ol, 13S-, O-TMS | 1.8424 | 0.006407 |
| l-Norleucine, N-ethoxycarbonyl-, decyl ester | 0.60649 | 0.042664 |
| Methyl 3,4-dimethoxyphenylhydroxyacetate, TMS derivative | 1.9183 | 0.044643 |
| Methyl 9-cis,11-trans-octadecadienoate | 2.2668 | 0.002531 |
| Naphthalene, 1-methyl- | 3.718 | 0.011475 |
| n-Nonadecanol-1 | 1.5084 | 0.011911 |
| Nonane, 2,6-dimethyl- | 1.8323 | 0.002771 |
| Octadecanamide, N-2-methylpropyl-N-nitroso- | 0.32678 | 0.005701 |
| Octane, 2,3,7-trimethyl- | 1.9888 | 0.000603 |
| Oleic Acid, Z-, TMS derivative | 1.5842 | 0.020916 |
| Oxalic acid, heptyl propyl ester | 0.66044 | 0.016778 |
| Phenol, 2,4-bis1-methylethyl-, acetate | 2.663 | 0.000517 |
| pm-4-Fluoroephedrine, N-trimethylsilyl-, trimethylsilyl ether | 0.53528 | 0.034911 |

| | | |
|---|---|---|
| pm-p-Methoxyamphetamine, N-trimethylsilyl- | 0.61052 | 0.02702 |
| Propylene glycol, 2TMS derivative | 3.0575 | 0.026156 |
| Propylparaben, TMS derivative | 3.3474 | 0.010665 |
| Pyrrole-2-carboxylic acid, 2TMS derivative | 0.65725 | 0.04564 |
| R-9-S-2-Hydroxymethylpyrrolidin-1-yl-3-methyl-3,4-dihydro-2H-benzob1,4,5oxathiazepine 1,1-dioxide | 4.7391 | 0.005459 |
| R---Phenylephrine, N-trimethylsilyl-, bistrimethylsilyl ether | 0.45322 | 0.011866 |
| Serine, 3TMS derivative | 0.43595 | 0.041998 |
| Stearic acid hydrazide | 3.1148 | 0.02159 |
| Sulfurous acid, isobutyl pentyl ester | 0.56612 | 0.008681 |
| trans-13-Octadecenoic acid | 0.34168 | 0.043301 |
| Tridecane, 4-methyl- | 1.6391 | 0.00429 |
| Trimethylsilylserine | 0.43595 | 0.041998 |
